# Toxicity of Ochratoxin to Early Life Stages of Zebrafish (*Danio rerio*)

**DOI:** 10.3390/toxins10070264

**Published:** 2018-06-28

**Authors:** Linda Tschirren, Seraina Siebenmann, Constanze Pietsch

**Affiliations:** Institute of Natural Resource Sciences (IUNR), Zurich University of Applied Sciences (ZHAW), Grüental, P.O. Box, CH-8820 Wädenswil, Switzerland; linda.tschirren@zhaw.ch (L.T.); siebeser@students.zhaw.ch (S.S.)

**Keywords:** mycotoxin, embryo toxicity, heart rates, oxidative stress

## Abstract

Ochratoxin A (OTA) is a known contaminant in fish feed but its effect on fish health remains rather unknown. A study was conducted to investigate the effects of different concentrations of ochratoxin on early life stages of zebrafish (*Danio rerio*). The tests with ochratoxin A showed a correlation between the exposure to mycotoxin and the amount of damage. The mortality rate and the incidents of embryonal damage was increased by increasing ochratoxin concentrations. The calculations resulted in a lethal concentration for 50% of the embryos (LC_50_) of 0.29 mg/L and a concentration at which 50% of the animals showed impairment (EC_50_) of 0.36 mg/L after 96 h of exposure. During the test, reduced heart rates were also observed revealing a clear dose-response relationship. The EC_50_ determination for this endpoint was 1.26 mg/L after 72 h of exposure. The measurement of oxidative stress was proven to be the most sensitive system to indicate OTA effects on the zebrafish embryos with an EC_50_ value of 0.067 mg/L after 72 h of exposure. The test validity was given because the control test with 3,4-Dichloroaniline (3,4-D) showed a LC_50_ value of 2.88 mg after 96 h of exposure which is comparable to the available reference values. According to the current knowledge, these experimental doses did not exceed the environmental concentrations of this ochratoxin A. However, this study raises concerns about the effects of ochratoxin on fish.

## 1. Introduction

Ochratoxin A (OTA) is mainly produced by fungi of the genus *Aspergillus* and *Penicillium.* As a consequence of their world-wide occurrence, OTA has been found in feed ingredients and feeds at variable contamination levels which are assumed to be caused by differences in to humidity and temperature during crop growth and during storage of feed ingredients and compounded feeds [1,2,3]. The contamination with OTA has to be taken seriously since OTA is assumed to be more stable in the environment than, for example aflatoxins [2,4]. Contaminated feed products lead to the introduction of OTA in the food chain and a risk for humans is assumed [5]. The presence of OTA in the food chain also resulted in detectable OTA levels in humans [6]. In addition, waste water is produced at different steps during wine production and winery effluents have been shown to contain considerably high concentrations of OTA [7,8] which might be an additional point source of OTA for aquatic environments. The effects of OTA in vertebrates are therefore of great concern.

In higher vertebrates, toxic effects of OTA are mainly observed in the kidney and liver and OTA was also reported to be teratogenic and immunotoxic [9]. In rodents, carcinogenic effects have also been observed [10]. In animals, therefore, a higher susceptibility to disease and more secondary infections have been observed [9] Embryotoxicity has been shown in amphibians and rats, mice, hamsters and chickens [11,12,13,14,15]. In addition, exposure of zebrafish embryos to OTA resulted in a variety of severe abnormalities, such as deformities, reduced growth and hatching rates and lethality at concentrations as low as 0.1 mg/L exposure medium [16]. Furthermore, the injection of rainbow trout with OTA resulted in kidney and liver damage and the calculation of a lethal concentration for 50% of the animals (LC_50_) after 96 h of exposure to OTA of 4.7 mg/kg body weight [17]. Dietary exposure of channel catfish (*Ictalurus punctatus*) and sea bass (*Dicentrarchus labrax*) to OTA led to reduced weight gains, poorer feed conversion rates, lower survival and changes of haematocrit values [5,18] in these fish species but not in Atlantic salmon (*Salmo salar* [19]). In addition, histopathological damage in the liver and posterior kidney and changes of immune parameters were observed in channel catfish [5,20,21]. Similar studies on Nile tilapia (*Oreochromis niloticus*) showed that increasing dietary OTA levels resulted in decreased growth, feed utilization and nutrient composition of the carcass [22]. In contrast to the investigations on fish, a study on shrimp reported no pronounced negative effects of OTA at levels of up to 1 mg/kg [23]. In naturally contaminated feeds, OTA commonly occurs together with other mycotoxins [24] but interactions with other toxins have not been reported.

The present study investigated effects of OTA to describe possible threshold values for fish embryo toxicity for this mycotoxin and targets of OTA in zebrafish.

## 2. Results

### 2.1. Effects on Zebrafish Embryo Development

**Figure 1:**
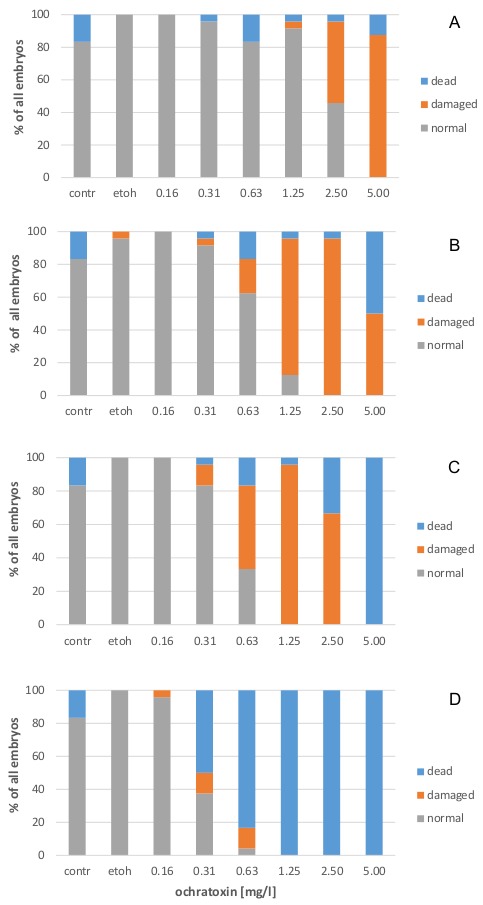
Concentration-dependent effects of OTA on zebrafish embryos after 24 (**A**), 48 (**B**), 72 (**C**) and 96 h (**D**) of exposure.

As expected, the embryos after 24 h of exposure still use the reserves from the yolk sac and do not yet have pigmentation. This exposure duration causes significant damage due to OTA exposure at concentrations higher than 1.25 mg/L (*p* = 0.000). The detrimental effects of OTA on zebrafish embryos followed a polynomic relationship after 24 h of exposure (y = 0.4595x^2^ + 18.254x − 1.6657; r^2^ = 0.97) and a concentration at which 50% of the embryos showed any detrimental effects (EC_50_ value) including death was identified at 2.65 mg/L (Figure 1A, Table 1). The calculation of a LC_50_ value at this point in time resulted in a high level with low reliability (LC_50_ = 24.22 mg/L; y = −0.0106x^2^ + 1.6564x + 3.6737; r^2^ = 0.21). After 48 h, body pigmentation in normal embryos starts; the eye is dark and the yolk sac is further depleted. However, the detrimental effects on the embryos led to a significant developmental retardation at concentrations higher than 0.31 mg/L (*p* = 0.011, Figure 1B) and the effects showed an EC_50_ value (integrating all detrimental effects on the embryos including death) of 0.73 mg/L (y = −28.917x^2^ + 121.81x − 23.178; r^2^ = 0.99) after 48 h of exposure. The single embryo that showed a conspicuous development in the solvent control had a yolk sac-oedema (Figure 1B), which was no longer detectable after 72 h and 96 h of exposure. At 48 h of exposure, a more reliable LC_50_ value of 2.57 mg/L OTA was obtained (y = 3.2147x^2^ − 7.919x + 8.3314, r² = 0.88). After 72 h of exposure, OTA concentrations higher than 0.31 mg/L resulted in significant damage to the embryos (*p* = 0.000, Figure 1C). At this point, an EC_50_ value (integrating all detrimental effects on the embryos including death) of 0.55 mg/L was calculated (y = −24.578x^2^ + 119.68x − 9.2249, r² = 0.95) and 50% of the animals were found to be dead at 3.32 mg/L OTA (y = 3.0667x^2^ + 4.2717x + 2.0125, r² = 0.98).

At 96 h of exposure, there was a pronounced increase in mortality. Significant effects on zebrafish development were observed at OTA concentrations higher than 0.16 mg/L (*p* = 0.000). At this point in time, an EC_50_ value (integrating all detrimental effects on the embryos including death) of 0.29 mg/L was calculated (y = −0.9341x^2^ + 2.2807x − 0.0455; r^2^ = 0.96), whereas the LC_50_ value occurred at 0.36 mg/L OTA (y = −88.12x^2^ + 198.92x − 9.9937, r² = 0.94). The ratio between the EC_50_ and LC_50_ values at each time point did not show a stable ratio (Table 1).

**Figure 2:**
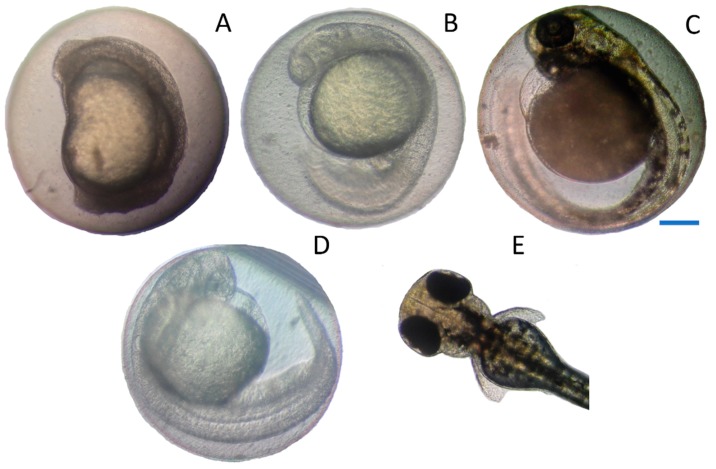
Zebrafish embryos after 24 h of exposure to OTA at a concentration of 5 mg/L (**A**) and after 72 h of exposure to 0.63 mg/L OTA (**B**) and to 0.31 mg/L OTA (**C**) showing the retardation of development compared with animals of normal development at 24 h (**D**) and the anterior part of a larvae hatched at 72 h (**E**); scale bar 200 μm.

Figure 2 shows embryos in different developmental stages with OTA treatment. After 24 h of exposure to 5 mg/L OTA, most embryos showed pronounced retardation of development. These embryos showed a developmental stage that should have been accomplished 16.5 ± 0.9 h (mean ± SEM) earlier according to a previous study ([25], Figure 2A). No further development of these embryos was observed at later points in time and all embryos treated with 5 mg/L OTA were dead after 72 h of exposure. The embryos treated for 24 h with 2.5 mg/L OTA and 1.25 mg/L OTA showed a growth retardation of 3.7 ± 1.3 h and 0.6 ± 0.6 h (means ± SEM), respectively, whereas all remaining fish showed no under-development at this time point. At 48 h of exposure, 92.2% of the embryos categorized as damaged embryos (Figure 1B) were underdeveloped. At 72 h of exposure, different levels of growth retardation were also observed at the lower OTA concentrations (Figure 2B,C). In total, 75.5% of all embryos listed in Figure 1C showed retardation of the development, whereas the 7% of the damaged embryos displayed effect on the blood circulation without showing under-development at the same time and the remaining embryos had oedema or had not yet hatched. As an example, the embryo shown in Figure 2B had not developed any pigmentation after 72 h of exposure to 0.63 mg/L OTA and showed a level of development comparable to a zebrafish embryo of less than 30 h after fertilization [25]. A high number of embryos showed deficiencies in blood circulation and the heart development. An example of this is an embryo is shown in Figure 2C that was treated with 0.31 mg/L OTA for 72 h.

While this embryo showed appropriate development of the head and tail and also possessed pigmentation of the eye and the remaining body, the yolk sac exhibited turbidity and most importantly, the heart appeared to be smaller and thinner with malformed chambers and the presence of a pericardial oedema and there was no heartbeat (Figure 2C).

The hatching occurred in the control and solvent control animal until 72 h of exposure (Table 2). The hatching success was significantly reduced upon exposure to 0.61 mg/L OTA or higher OTA concentrations (*p* = 0.000). The inhibition of hatching reached an EC_50_ value of 0.44 mg/L OTA at 72 h of exposure (y = 52.936x^2^ − 154.9x + 108.14, r² = 0.92) and an EC_50_ value of 1.76 mg/L OTA after 96 h of exposure (14.583x^2^ − 106.25x + 191.67, r² = 1.00). At concentrations of 1.25 mg OTA or higher no embryo was hatching.

### 2.2. Heart Rates

**Figure 3:**
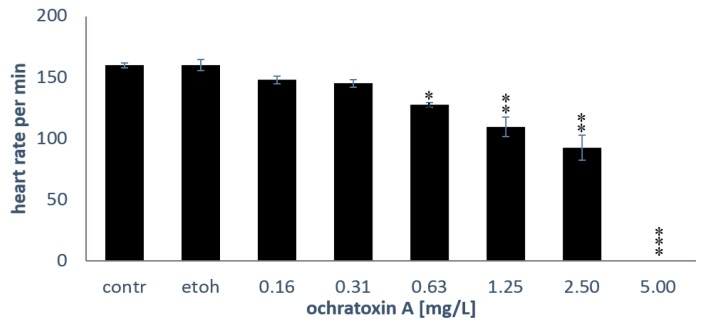
Heart rate of embryos. The mean ± SEM of six embryos is shown after 48 h of exposure; the asterisks (*: *p* < 0.05; **: *p* < 0.01; ***: *p* < 0.001) indicate significant differences to the solvent control (etoh), Mann-Whitney U-tests with Bonferroni corrections for multiple comparisons, *n* = 6 embryos per treatment.

After 48 h of exposure, heartbeats of the embryos were assessed (Figure 3). The solvent ethanol had no effect on the heart rates in the zebrafish embryos.

**Figure 4:**
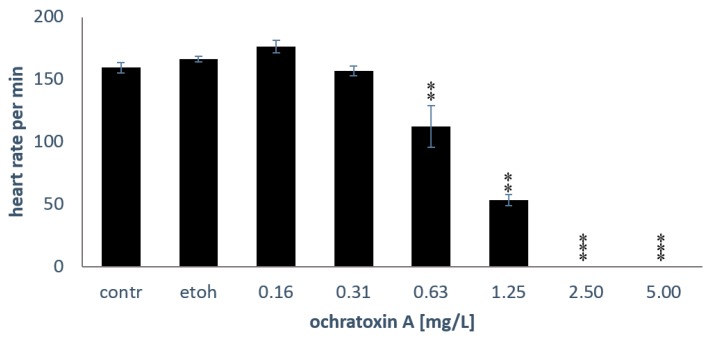
Heart rate of embryos. The mean ± SEM of six embryos is shown after 72 h of exposure; the asterisks (**: *p* < 0.01; ***: *p* < 0.001) indicate significant differences to the solvent control (etoh), Mann-Whitney U-tests with Bonferroni corrections for multiple comparisons, *n* = 6 embryos per treatment.

Figure 4 shows that the heart rate per minute was also significantly reduced in embryos exposed to OTA for 72 h (*p* = 0.002; EC_50_ of 1.26 mg/L, y = −20.353x^2^ − 74.484x + 176.56; r^2^ = 0.96). After 72 h of exposure, no embryo exposed to 2.5 mg/L OTA or higher showed any heartbeat.

### 2.3. Oxidative Stress

**Figure 5:**
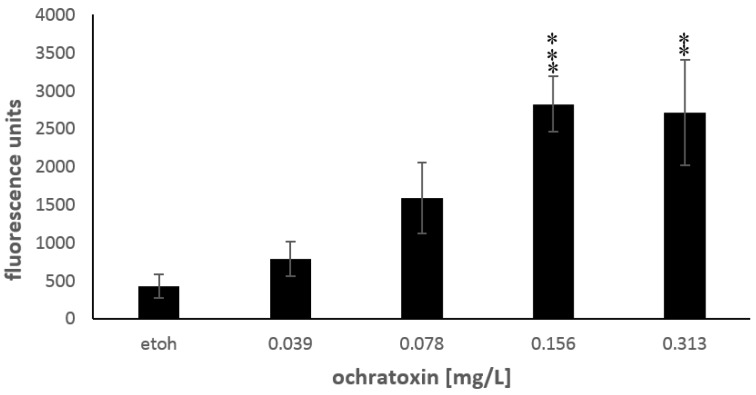
Concentration-dependent effects of OTA on oxidative stress in zebrafish embryos at 72 h of exposure displayed as fluorescence units emitted by the cell-permeant dye 2′,7′-dichlorodihydrofluorescein diacetate (H_2_DCF-DA) upon excitation at 480 nm; the asterisks (**: *p* < 0.01; ***: *p* < 0.001) indicate significant differences to the solvent control (etoh), Mann-Whitney U-test with Bonferroni corrections for multiple comparisons, *n* = 8–14 embryos per treatment.

The measurement of oxidative stress in zebrafish embryos showed a significant increase of the emitted fluorescence units with increasing OTA concentrations (*p* = 0.000, Figure 5) which were found to be significantly different from the solvent control-treated embryos at concentrations of more than 0.078 mg/L OTA. The measurements of oxidative stress yielded an EC_50_ value of 0.067 mg/L OTA (y = −46890x^2^ + 22883 x + 181.71; r^2^ = 0.96).

### 2.4. 3,4-Dichloroaniline

**Figure 6:**
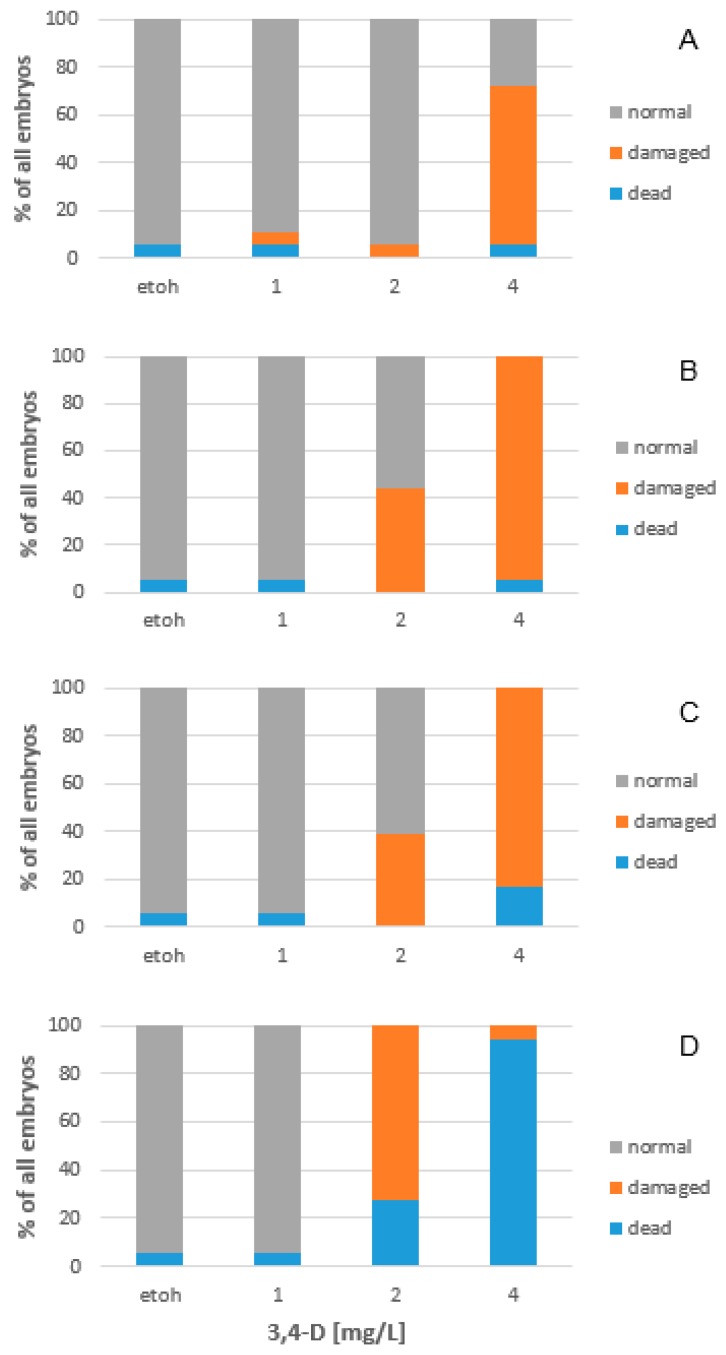
Concentration-dependent effects of 3,4-D on zebrafish embryos after 24 (**A**), 48 (**B**), 72 (**C**) and 96 h (**D**) of exposure.

Zebrafish embryos were sensitive to 3,4-Dichloroaniline (3,4-D) and showed impairment of development (Figure 6). The respective LC_50_ and EC_50_ values are displayed in Table 3.

As expected, the exposure to the reference compound 3,4-D resulted in mortality of the fish embryos (Table 3). After 24 h of exposure, significant damage occurred in embryos that had been treated with 4 mg/L 3,4-D (*p* = 0.000) but no significant increase of lethal damages. A LC_50_ value of 9.06 mg/L OTA was noted at this time point. After 48 h of exposure, damaged embryos occurred resulting in an EC_50_ value of 2.09 mg/L (y = 19.444x^2^ − 19.444x + 5.5556, r² = 1.00), whereas an LC_50_ value of 9.06 mg/L (y = 1.0101x^2^ − 4.3434x + 6.4646, r² = 0.56) was noted. Exposure of the embryos to 3,4-D for 72 h and 96 h further decreased the EC_50_ and LC_50_ values (Table 3).

Compared to OTA the reference compound 3,4-D caused different damages in the embryos. At 24 h, the exposure to 3,4-D resulted in underdeveloped embryos (18.8% of the damaged embryos) and deformations of embryos (75% of the damaged embryos showing mainly missing eyes and deformations of the head). Only one damaged embryos showed underdevelopment and deformations. At 48 h of exposure to 3,4-D, 34.6% of the embryos listed as damaged in Figure 6 showed yolk sac oedema and 46.2% of the embryos showed deformations and 19.2% of the impaired embryos showed both, oedema and deformations. The incubation of the embryos for 72 h with 3,4-D, 38.1% of the embryos listed as damaged in Figure 6 showed yolk sac oedema and 42.9% of the embryos showed deformations, whereas 19.0% of the impaired embryos showed both, oedema and deformations. At this time point, only 11.1% of all embryos showed impairment of the heart beating. At 96 h of exposure 92.8% of the impaired but still alive embryos showed oedema and only 7.1% deformations. The hatching success was significantly reduced in embryos treated with 4 mg/L 3,4-D after 72 h of exposure and significantly impaired by exposure to 2 mg/L 3,4-D after 96 h of exposure.

## 3. Discussion

### 3.1. Toxicity of OTA to Fish Embryos

Based on previous studies [26,27], the LC_50_ for OTA in different higher vertebrate species ranges from 2 and 58 mg/kg body weight. However, fish species appear to be more sensitive to OTA than higher vertebrates. If this is also true for the different structures of OTA (i.e., other ochratoxins or of their metabolites [28] remains unknown. The LC_50_ in adult seabass (*Dicentrarchus labrax* L.) after oral exposure was found to be at 9.23 mg OTA per kg diet after 96 h of exposure which was calculated to be equal to an exposure value of 0.28 mg/kg body weight [18]. Investigations on rainbow trout showed a LC_50_ of 5.53 mg OTA kg^−1^ body weight in this fish species after intraperitoneal injection [17]. A study on the embryo toxicity in zebrafish yielded even lower LC_50_ values [16]. However, this study did not indicate the possible toxicological mechanisms that might be involved in OTA toxicity in zebrafish embryos. Therefore, low doses of OTA were again used in the present study to calculate more accurate LC_50_ values and describe the targets of OTA in fish embryos more in detail.

Increasing OTA concentrations clearly resulted in increased mortality of zebrafish embryos. Concentrations higher than 0.63 mg/L led to 100% mortality after 96 h of exposure. A LC_50_-Wert value of 0.36 mg/L OTA was calculated after 96 h of exposure. Different effects on the zebrafish embryos were observed at even lower OTA concentrations and an EC_50_ value of 0.29 mg/L was obtained. Most of the embryos treated with OTA concentrations higher than 1.25 mg/L showed early retardation of development. In addition, it was observed that especially blood circulation and the heart development and heart rate were negatively affected by OTA exposure. In previous studies, cardiac abnormalities due to OTA exposure have only been described in rats and chickens [29,30]. Besides these effects, embryos exposed to lower OTA concentration more often showed yolk sac oedema and died at later points in time.

The present study showed higher EC_50_ and LC_50_ values than previously reported [16] and since especially the values for 24 h of exposure were found to be higher than those described by others [16]. However, the study of Haq and the co-authors [16] used no independently incubated embryos but reared 5 animals together in one replicate for their incubations and did not correct for multiple comparisons between the different treatments. Due to these experimental flaws, the present study is assumed to yield more realistic information on significant threshold levels for OTA toxicity in zebrafish. What is also noticeable when comparing the present study and the previous report [16] is that the present experiments yielded less deformations of the embryos. Moreover, the previously observed factor of 10 between LC_50_ and EC_50_ [16] was not confirmed by the present study. The reason for this might be the start of exposure that started at <2 h post fertilization in the study conducted by Haq et al. [16] but at 3 h post fertilization in the present study.

OTA has been described as a frequent contaminant of diverse food and feed ingredients and can also be detected in processed animal feeds [1,2,3]. Maximum allowable levels for OTA have been established in Europe (20 μg/kg) and also in other regions [31]. The concentrations in finished animal feeds are often low but might be detrimental for fish if highly contaminated ingredients are used for feed production. High concentrations of OTA have been found in commonly used ingredients of fish feed in some cases, for example in corn (up to 1850 μg/kg, [32]), wheat (up to 1024 μg/kg, [33]) soybean and sunflower products (up to 350 and 240 μg/kg, respectively [32]). In addition, inappropriate storage conditions for 6 weeks may lead to considerable amounts of OTA in commercial fish feeds (up to 400 μg/kg, unpublished results of the authors). In addition, approximately 0.090 mg/L OTA have been reported in effluents from wine production and the time for biodegradation was relevant for the present study [8]. The present study, together with previous studies [16,18], demonstrated that even very low amounts of this mycotoxin can have detrimental effects on fish.

### 3.2. Detection of Reactive Oxygen Species (ROS)

The measurement of oxidative stress in the zebrafish embryos showed a very low EC_50_ value of 0.067 mg/L OTA after 72 h of exposure. The detection of ROS in the embryos is therefore a very sensitive method to confirm that OTA is detrimental for zebrafish embryos. Oxidative stress has also been observed in OTA-treated rodents [34,35,36], although a link between ROS occurrence and detrimental effects, for example, renal toxicity was found to be low in an additional study on rats [37]. The mechanism by which OTA is able to increase ROS formation has not been fully explored so far. One possible explanation for increased ROS production and depletion of intracellular antioxidants in cells [38] might be the formation of a phenoxyl radical from OTA by peroxidases [39]. The presence of glutathione may lead to reconversion of the phenoxyl radical to OTA which generated a superoxide anion radical [40]. Superoxide anion radicals can lead to further oxidative stress by forming hydrogen peroxide and possible induction of a Fenton reaction and the subsequent formation of hydroxyl radicals resulting in further oxidative damage. Oxidative stress due to OTA exposure has been reported to lead to damage to lipids, proteins and DNA [36].

### 3.3. Positive Controls

The present study used 3,4-Dichloroaniline (3,4-D) as a positive control and at a concentration of 2.88 mg/L, a mortality of 50% was observed after 96 h of exposure. This 3,4-D concentration therefore fulfilled the criteria for the early life stage test based on the revised OECD guideline [41]. In addition, the observed effects of 3,4-D on the embryos was comparable to the effects on survival and development in fish embryos and larvae [42,43], although the zebrafish embryos in the present study showed less pronounced effect on the heart function and the skeletal development than the early life stages of the rare minnow (*Gobiocypris rarus*, [43]).

## 4. Conclusions

OTA strongly interferes with the development of the early life stages of zebrafish. The toxicity assays revealed that OTA induced dose-dependent mortality in zebrafish embryos resulting in a LC_50_ value of 0.36 mg/L after 96 h of exposure. This confirms that OTA has detrimental effect on fish. The EC_50_ was calculated to be 0.29 mg/L for damage in the embryos. In addition, a correlation between the OTA concentrations and the decrease of the heart rates was observed. The assay for ROS production in OTA-treated embryos showed increased oxidative stress in treatments higher than 0.078 mg/L and was the most sensitive endpoint for detrimental effects of OTA in zebrafish embryos. The assays conducted indicate that OTA-related production of ROS contributed to the detrimental effects of this mycotoxin on zebrafish embryos. This indicates that the detection of ROS can be a useful tool to detect cellular influences of OTA on fish before potential morphological impairment occurs. However, the exact cellular mechanism(s) of action of OTA on cellular functions in different organs still remains to be investigated and further research is needed to provide an organ-wide description of possible effects of this mycotoxin.

## 5. Materials and Methods

### 5.1. Chemicals

All chemicals were obtained from Sigma-Aldrich (Buchs, Switzerland) unless indicated otherwise. The OTA (Sigma Cat. No. 01877; produced by *Petromyces albertensi*, lot No. 067M4011V) and 3,4-Dichloroaniline (Sigma Cat. No. 437778; lot No. 13509KQV) were solubilized in pure ethanol before use.

### 5.2. Preparation of Exposure Medium

ISO water [44] containing calcium chloride 2-hydrate (294 mg/L), magnesium sulfate-7-hydrate (123.3 mg/L), sodium hydrogen carbonate (63 mg/L) and potassium chloride (5.5 mg/L) was adjusted to a pH of 7.4, sterile filtered and adjusted to a temperature of 27 °C before use. OTA was solubilized in pure ethanol and added to the ISO medium at serial concentrations ranging from 5 mg/L to 0.039 mg/L (leading to a final ethanol concentration of 0.1% ethanol).

### 5.3. Exposure of Fish Embryos

The zebrafish eggs were obtained from the EAWAG (Dübendorf, Switzerland), whereas the ROS measurements were performed with zebrafish eggs obtained from the ZHAW brood stock that originated from EAWAG zebrafish adults. The test was conducted according to the DIN norm 38415-6 [44]. For each exposure concentration, 18–24 eggs were exposed and negative controls containing ethanol as a solvent were included. The solvent concentration did not exceed 0.1% in each of the treatments. The eggs were incubated at 27 °C and a light/dark cycle of 16h:8h in a Multitron Pro incubator (Infors AG, Bottmingen, Switzerland). The eggs were incubated with different ochratoxin concentrations at 3 h post fertilization in sterile ISO water containing MgSO_4_, CaCl_2_, NaHCO_3_ and KCl at a pH of 7.4. As a positive control, 3,4-Dichloroaniline (3,4-D) was used at concentrations between 1 and 4 mg/L as a positive control, since it was recommended by the revised OECD guideline as a new criterion for early life stage tests with zebrafish at a concentration of 4 mg/L of this substance resulting at least in a mortality of 30% after 96 h of exposure [41]. Determination of development of all embryos using a microscope (Leica Type 090-135.006, Leica Microsystems (Switzerland) AG, Heerbrugg, Switzerland) was conducted at 24, 48, 72 and 96 h post fertilization.

### 5.4. Assessment of Development

For the experiment 4 plates with 4 embryos for each treatment were incubated for 96 h at 27 °C whereby each plate contained the respective control exposures with eggs exposed to ISO water only and a solvent control containing the same ethanol content as the ochratoxin treatments (0.1%). For the subsequent experiment evaluating ROS, 4 plates with 6 embryos for each treatment were incubated for 72 h at 27 °C. Mortality, developmental stage, including the presence of the eyes, somites and the movement of the tail and possible occurrence of oedema (Table 4) were noted after 24 h of exposure using a microscope (Leica-Microscope Type 090-135.006, Leica Microsystems (Switzerland) AG, Heerbrugg, Switzerland). After 48 h and 72 h, pigmentation and blood flow were also assessed. Exact staging of the embryos was done according to Braunbeck and Lammer [25]. A dead embryo was noted if the material in the egg appeared to be at various stages of decomposition or coagulated. At the time points 48 and 72 h post fertilization the heartbeats of each living embryo were assessed for 20 to 30 sec manually under the microscope and heartbeats per minute were calculated.

### 5.5. Measurement of Reactive Oxygen Species (ROS)

After 72 h of exposure, ROS production was measured using the fluorescent dye 2′,7′-dichlorodihydrofluorescein diacetate (H_2_DCF-DA) as has previously been done in cell cultures of mammals and fish [45,46]. After exposure to OTA, the embryos were killed by an overdose MS-222 (150 mg/L in NaHCO_3_-buffered ISO medium), transferred to an opaque, flat-bottom 96-well plate and the medium was replaced by 100 μL of H_2_DCF-DA solution (final concentration: 5 μM in ISO medium) was added to each well. The plate was incubated for 30 min at room temperature in the dark. Thereafter, fluorescence at excitation and emission wavelengths of 480 nm and 535 nm, respectively, was measured with a plate reader (Infinite M200, Tecan Instruments, Männedorf, Switzerland).

### 5.6. Calculation of the 50% Level for Effects and Lethality

The half maximal effective concentration (EC_50_) was determined by plotting the responses of the embryos (including death) against the test concentrations. The relationship between the concentrations and the resulting effects followed polynomic equations which are reported for each duration of exposure separately including the respective correlation coefficients. For damages such as deformations and the occurrence of oedema, calculations of the concentration at which 50% of the effects were observed were conducted separately from the heart beat calculations and the measurements of oxidative stress. The concentration at which 50% of the embryos were found to be dead (LC_50_) was calculated similarly.

### 5.7. Statistics

Chi-square statistics were employed to compare the incidence of damage to the embryos by using the Monte Carlo approximation (using 10,000 simulations, with confidence interval of 99%) to the Pearson chi-square Test. The heartrates and fluorescence units were compared by using Mann Whitney U-tests and Kruskal Wallis tests (SPSS version 24 for Windows; SPSS Inc., Chicago, IL, USA). Differences between treatment groups were considered statistically significant when *p* < 0.05 to which the according Bonferroni corrections for multiple comparisons were applied.

## Figures and Tables

**Table 1:** Summary of the LC_50_ and EC_50_ values for the respective test durations for embryos exposed to ochratoxin, *n* = 24 for each OTA concentration and the solvent control.

Time Point	EC_50_ (mg/L)	LC_50_ (mg/L)	Ratio LC_50_ to EC_50_
24 h	2.65	24.22	9.14
48 h	0.73	2.57	3.52
72 h	0.56	3.32	5.93
96 h	0.29	0.36	1.24

**Table 2:** Summary of the hatching success for the respective test durations reported for all exposed embryos in the different treatments (*n* = 24 for each) and displayed as the percentage of the embryos still alive at this time point in brackets.

Treatment	72 h	96 h
contr	83.3% (100%)	83.3% (100%)
etoh	95.8% (95.8%)	100% (100%)
0.16 mg/L OTA	95.8% (95.8%)	100% (100%)
0.31 mg/L OTA	79.2% (82.6%)	37.5% (81.8%)
0.63 mg/L OTA	16.7% (21.1%)	4.2% (33.3%)
1.25 mg/L OTA	0% (0%)	0% (0%)
2.50 mg/L OTA	0% (0%)	0% (0%)
5.00 mg/L OTA	0% (0%)	0% (0%)

**Table 3:** Summary of the LC_50_ and EC_50_ values for the respective test durations for embryos exposed to 3,4-Dichloroaniline (3,4-D), *n* = 18 for each 3,4-D concentration and the solvent control.

Time Point	EC_50_ (mg/L)	LC_50_ (mg/L)	Ratio LC_50_ to EC_50_
24 h	3.47	9.06	2.61
48 h	2.09	9.06	4.59
72 h	2.22	6.08	2.74
96 h	1.59	2.88	1.81

**Table 4:** Endpoints used for assessment.

Time Point	Endpoint
24 h	eye visible, somites, tail movements
48 h	pigmentation, blood circulation, heartbeats, oedema, embryo movements
72 h	pigmentation, blood circulation, heartbeats, oedema, embryo movements, hatching
96 h	pigmentation, blood circulation, heartbeats, oedema, embryo movements, hatching
